# Protection of ischemic post conditioning against transient focal ischemia-induced brain damage is associated with inhibition of neuroinflammation via modulation of TLR2 and TLR4 pathways

**DOI:** 10.1186/1742-2094-11-15

**Published:** 2014-01-24

**Authors:** Ying Wang, Pengfei Ge, Li Yang, Chunyun Wu, Hao Zha, Tianfei Luo, Yuhong Zhu

**Affiliations:** 1 Department of Neurology, Second Affiliated Hospital of Kunming Medical University, Kunming 650031, China; 2 Department of Neurosurgery, First Hospital of Jilin University, Changchun 130021, China; 3 Department of Histology and Embryology, Kunming Medical University, Kunming 650031, China; 4 Department of Neurology, First Hospital of Jilin University, Changchun 130021, China

**Keywords:** Ischemic postconditioning, Cerebral ischemia/reperfusion, TLR2, TLR4, Neuroinflammation

## Abstract

**Background and purpose:**

Ischemic postconditioning has been demonstrated to be a protective procedure to brain damage caused by transient focal ischemia/reperfusion. However, it is elusive whether the protection of postconditioning against brain damage and neuroinflammation is via regulating TLR2 and TLR4 pathways. In the present study, we examined the protection of ischemic postconditioning performed immediately prior to the recovery of cerebral blood supply on brain damage caused by various duration of ischemia and tested the hypothesis that its protection is via inhibition of neuroinflammation by modulating TLR2/TLR4 pathways.

**Methods:**

Brain damage in rats was induced by using the middle cerebral artery occlusion (MCAO) model. Ischemic postconditioning consisting of fivecycles of ten seconds of ischemia and reperfusion was performed immediately following theischemic episode Theduration of administration of ischemic postconditioning was examined by comparing its effects on infarction volume, cerebral edema and neurological function in 2, 3, 4, 4.5and 6 hour ischemia groups. The protective mechanism of ischemic postconditioning was investigated by comparing its effects on apoptosis, production of the neurotoxic cytokine IL-1β and the transcription and expression of TLR2, TLR4 and IRAK4 in the 2 and 4.5 hour ischemia groups.

**Results:**

Ischemic postconditioning significantly attenuated cerebral infarction, cerebral edema and neurological dysfunction in ischemia groups of up to 4 hours duration, but not in 4.5and 6 hour ischemia groups. It also inhibited apoptosis, production of IL-1β, abnormal transcription and expression of TLR2, TLR4 and IRAK4 in the 2 hour ischemia group, but not in the 4.5 hour ischemia group.

**Conclusions:**

Ischemic postconditioning protected brain damage caused by 2, 3 and 4 hours of ischemia, but not by 4.5 and 6 hours of ischemia. The protection of ischemic postconditioning is associated with its inhibition of neuroinflammation via inhibition of TLR2 and TLR4 pathways.

## Introduction

Cerebral damage due to reperfusion following ischemia has been proven to be an important factor affecting the prognosis of revascularization of occluded blood vessels [1]. Thus, many researchers focus on developing new chemicals or methods to prevent brain injury caused by ischemia and reperfusion. Recently, accumulating evidence from animal studies and clinical trials has shown that ischemic postconditioning is an effective procedure to suppress secondary tissue injury following recovery of blood supply. Ischemic postconditioning is defined as a series of rapid intermittent interruptions of blood flow in the early phase of reperfusion that mechanically alters the hydrodynamics of reperfusion [2]. Ischemic postconditioning has been found to protect ischemia/reperfusion-induced tissue injury in brain, as well as in heart, liver and intestine [3-5]. Wang *et al*. and Ren *et al*. respectively have reported that ischemic postconditioning protected rat cerebral injury caused by reperfusion following either global ischemia or focal ischemia [6,7]. Therefore, ischemic postconditioning, as an emerging protective method, might be used clinically to prevent tissue damage caused by reperfusion due to revascularization of occluded blood vessels.

TLRs (Toll-like receptors) are the main signal pathways responsible for regulating endogenous or exogenous inflammation [8]. Neuroinflammation mediated by TLR2 or TLR4 was proved to play an active role in aggravating brain damage caused by ischemia/reperfusion. Under ischemic stimuli, TLR2 and TLR4 are both found to be expressed on neurons and glial cells such as microglia and astrocytes and would be activated when they are attached to their corresponding ligands such as heat-shock proteins (HSPs) and high mobility group box 1 (HMGB1) [9]. By contrast, inhibition of TLR2 or TLR4 pathway was reported to produce neuroprotection. Lehnardt *et al*. found that TLR2-deficient mice develop a decreased CNS injury compared to wild type mice insulted by focal cerebral ischemia [10]. Similarly, Ahmad *et al*. found that the expressional levels of neurotoxic cytokines TNF-α and IL-1β induced by traumatic brain injury was mitigated in TLR4 knockout mice [11]. Therefore, these studies showed that inhibition of TLR2- or TLR4- mediated neuroinflammation would exert protection on ischemia/reperfusion-induced brain damage.

Recently, ischemic postconditioning has been found to inhibit ischemia/reperfusion-induced inflammation in brain, heart, lung and liver [12-14]. Kong *et al*. reported that ischemic postconditioning suppressed the abnormal expression of inflammation mediators such as IL-1β and IL-6 caused by cerebral ischemia and reperfusion [15]. Joo *et al*. showed that the protection of ischemic postconditioning was associated with inhibition of the reduction in immune cell numbers in the peripheral blood caused by cerebral ischemia/reperfusion [11]. Also, Wu *et al*. found that ischemic postconditioning reduced post-ischemic release of the cytokines TNF -α and IL-8 [12]. Therefore, it remains elusive as to whether the inhibitory effect of ischemic postconditioning on inflammation is via modulating the TLR2/TLR4 pathway. Additionally, despite the effects of various ischemic postconditioning strategies that have been studied widely, the time duration of ischemic postconditioning treatment remains unclear. Thus, we investigated these two issues in this study by using the rat middle cerebral artery occlusion (MCAO) model.

## Materials and methods

### Animals

Adult male Sprague–Dawley rats (*n* = 104; weighing 280 to 300 g; seven to eight weeks of age), purchased from Chengdu Dashuo Biotechnological Company (Chengdu, China), were housed in a temperature-controlled room (22 to 25°C) on a 12-hour light/dark cycle with free access to food and water. All animal procedures were approved by the ethical committee for animal experiments, Kunming Medical University. All possible measures were taken to reduce animal suffering and numbers of animals in this study.

### Animal groups and surgical procedure

At the start of the study, the rats were randomly assigned into a sham-operated group, an ischemia group and an ischemic postconditioning group according to a computer generated randomization schedule.

Cerebralischemia was produced by using the middle cerebral artery occlusion (MCAO) model in rats as previously described [7]. In brief, a surgical incision was made to expose the right common carotid artery (CCA), internal carotid artery and external carotid artery. The proximal CCA then was ligated, and an occlusion filament was inserted into the internal carotid artery through the CCA 19 to 21 mm distal from the bifurcation to occlude the origin of the middle cerebral artery (MCA). After induction of ischemia, the filament was withdrawn and the rats were placed into a cage to recover from anesthesia at room temperature, with free access to food and drink.

The rats in the ischemia group were subjected to 2, 3, 4 h, 4.5, and 6 hours of focal cerebral ischemia. Ischemic postconditioning was performed at the beginning of reperfusion, and the ischemic postconditioning rats were subjected to five cycles of ten seconds of clipping both carotid arteries and tenseconds of reperfusion. In the sham group, rats were subjected to the same procedures except for occlusion of the MCA.

### Measurement of infarct volume and cerebral edema

At reperfusion at 48 hours, six rats from each group were chosen randomly, decapitated and the brains were rapidly removed. Brains were cut into 2-mm-thick coronal sections and stained with 1% triphenyl tetrazolium chloride (confirm) (TTC) solution for 30 minutes at 37°C followed by overnight immersion in 4% paraformaldehyde. The infarct volume and cerebral edema were calculated as previously described [16] using the formulae:

$$\begin{array}{ll} \text{Infarct}\;\text{volume}\;\left(\%\right)= & \left[\left(\text{Volume}\;\text{of}\;\text{the}\;\text{normal}\;\text{hemisphere}\right)\right)\\ & \;-\text{non}‒\text{infarct}\;\text{volume}\;\text{of}\;\text{the}\;\text{infarct}\;\\ & \;\left(\text{hemisphere}\right)/\text{Volume}\;\text{of}\;\text{the}\\ & \;\left(\;\text{normal}\;\text{hemisphere}\right]\times 100\% \end{array}$$

$$\begin{array}{ll} \text{Relative}\;\text{cerebral}\;\text{edema}\;\left(\%\right)= & \left[\left(\text{Volume}\;\text{of}\;\mathrm{the}\;\text{infarct}\;\text{hemisphere}\right)\right)\\ & \left(\;-\mathrm{volume}\;\mathrm{of}\;\text{the}\;\text{normal}\;\text{hemisphere}\right)\\ & \left(\;/\text{Volume}\;\mathrm{of}\;\text{the}\;\text{infarct}\;\text{hemisphere}\right]\\ & \;\times 100\% \end{array}$$

### Evaluation of neurological functional score

Neurological functional scores were evaluated at 48 hours post reperfusion. The test consists of two aspects of neurological function as has been previously described [17]: (1) the postural reflex test to examine upper body posture while the animal is suspended by the tail; (2) the forelimb placing test to examine sensorimotor integration in forelimb placing responses to visual, tactile, and proprioceptive stimuli. Neurological function was graded on a scale of 0 to 12 (normal score, 0; maximal score, 12).

### Analysis of apoptosis by flow cytometry

At 48 hours post reperfusion, four rats from each group were deeply anesthetized with chloral hydrate, and the tissues from the infarcted cortex were collected and maceratedmechanically in ice-cold PBS buffer before being filtered with a 30 μm pore size nylon filter. After the cellular number was adjusted to 1 × 10^6^/ml, the cells were centrifuged for ten minutes at 2,000 rpm, washed three times with PBS and fixed for 30 minutes in 70% ethanol. The fixed cells were washed three times with PBS, and suspended in PBS containing 0.1% Triton X-100 and 0.2% BSA at 37°C for 15 minutes, followed by being washed with PBS containing 0.2% BSA. After the cells had been incubated for one hour at 37°C with 30 μl FITC-dUTP (Roche Appiled Science, Mannheim, Germany), they were treated with 20 μl RNAse (10 mg/ml) at 37°C for 15 minutes and 500 μl of propidium iodide (100 μg/ml) for 30 minutes in the dark. The cells were then analyzed using flow cytometry (Beckman 4CR) and the rate of apoptosis was analyzed using software WinCycle DNA (Phoenix Flow Systems, San Diego, CA, USA).

### Immunohistochemistry

At 48 hours post reperfusion, four rats from each group were deeply anesthetized with chloral hydrate and perfused transcardially with 0.9% saline, followed by 4% paraformaldehyde in 0.1 mmol/L PBS. The brain was removed, fixed overnight in 4% paraformaldehyde in 0.1 M PBS, dehydrated in a graded series of alcohols, and embedded in paraffin. The embedded brains were then cut coronally into 4 μm-thick sections, incubated overnight at 4°C with 1:100 rabbit anti-TLR2 (Boster Biotechnology Company, Wuhan, China) and 1:100 mouse anti-TLR4 (Abcam, Cambridge, USA). Staining was developed with nickel-DAB solution (Maixin Biotech Company, Fuzhou, China). The positive cells were counted under a 400 × light microscope in five visual fields of the ischemic cortex region of the infarcted area and the images were captured by a digital camera connected to a microscope (Olympus BX43, Japan).

### Double immunofluorescence staining

The paraffin-embedded brain was serially cut into 10 μm-thick sections in coronal plane. After the standard histological procedures, sections were incubated overnight at 4°C in the mixture of 1:100 rabbit anti-TLR2 (Boster Biotechnology Company, Wuhan, China)/1:100 mouse anti-NeuN (Millipore Corporation, Billerica, USA), 1:100 rabbit anti-TLR2/1:1,000 mouse anti-GFAP (Millipore Corporation, Billerica, USA), 1:100 rabbit anti-TLR2 (Boster Biotechnology Company, Wuhan, China), 1:100 rabbit anti-TLR4 (Boster Biotechnology Company, Wuhan, China)/1:100 mouse anti-NeuN (Millipore Corporation, Billerica, USA), 1:100 mouse anti-TLR4 (Abcam, Cambridge, USA)/1:1,000 rabbit anti-GFAP (Millipore Corporation, Billerica, USA), 1:100 mouse anti-TLR4 (Abcam, Cambridge, USA). Then the sections were incubated respectively with 1:800 Alexa Fluor 568 donkey anti-rabbit (Invitrogen Life Technologies, NY, USA), 1:300 Alexa Fluor 555 donkey anti-mouse (Beyotime) for one hour at room temperature, followed incubated with 1:800 Alexa Fluor 488 donkey anti-mouse (Invitrogen Life Technologies, NY, USA), 1:800 Alexa Fluor 488 donkey anti-rabbit (Invitrogen Life Technologies, NY, USA), 1:100 Lectin-FITC conjugate (Sigma-Aldrich, St. Louis, USA) for onehour at room temperature. Finally, these sections were observed under laser confocal microscope (Leica, Germany).

### Real-time PCR

At 48 hours post reperfusion, the tissues from the infarcted cortex of four rats in each group were collected and the mRNA expressions of TLR2, TLR4, IRAK4 and IL-1β were assayed by using real-time RT-PCR. In brief, total RNA of infarcted cortex was isolated by Trizol reagent. Secondly, RNA was quantified by measuring the optical density at 260/280 nm, and adjusted RNA concentration of each sample to 50 ng/μL. The SYBR^®^ green DNA PCR Master Mix (Fermentas, Ottawa, Canada) was used for the real-time PCR analysis. The nucleotide sequences of primers used in this experiment were designed according to the sequence described as follows:

TLR2 (F: AAGTAGAAACGGTAACAATACGGAG; R: GTCGGGCATAGGCTGAAAAC, 368 bp);

TLR4 (F: AAAACCTAAGGAGAGGAGGCTAA;

R: AAAAAAAGAAACTGACAGAAAATGG, 248 bp);

IRAK4 (F: GCACGAGAAGAAAAACAGACGG; R: ACAAACGCAAACAATAGGCAAG, 170 bp);

IL-1β (F: ATCCCAAACAATACCCAAAGAAG; R: GAACTGTGCAGACTCAAA CTCCA, 95 bp);

GAPDH (F: ACGGCAAGTTCAACGGCACAG; R: GACGCC AGTAGACTCCACGACA, 146 bp)(Ok). Relative abundance of mRNA was calculated after normalization to glyceraldehyde 3-phosphate dehydrogenase (GAPDH) RNA. The relative quantification was determined using the comparative CT method [18].

### Western blotting

At 48 hours post reperfusion, the tissues from infarcted cortex of four rats in each group were homogenized using a total protein extraction kit (Beyotime Institute of Biotechnology, Nanjing, China). The homogenates were then centrifuged at 10,000 g for ten minutes at 4°C, and the protein concentration of the supernatants was measured. Western blot analysis was conducted with 10% sodium dodecyl sulfate-polyacrylamide gel electrophoresis (SDS-PAGE). Four samples (from four different rats) in every experimental group were used for statistical analysis. After electrophoresis, proteins were transferred onto PVDF membranes (Millipore Corporation, Billerica, USA). The membranes were probed with anti-TLR4 (1:1,000, Boster Biotechnology Company, Wuhan, China), anti-TLR2 (1:1,000, Epitomics), anti-IRAK4 (1:1,000, Cell signaling), anti-IL-1β (1:1,000, Proteintech), anti-β-actin (1:2,000, Proteintech). The membranes were then incubated with goat anti-rabbit horseradish peroxidase-conjugated secondary antibody (1:5,000) for one hour at room temperature. The blots were developed with the enhanced chemiluminescence (ECL) detection method, and the relative density of bands was analyzed with a chemiluminescence system (Bio-Rad Laboratories, Philadelphia, USA).

### Statistical analyses

All data are expressed as mean ± SD and are suitable for analysis using nonparametric tests. The Kolmogorov-Smirnov test was applied to test for a normal distribution. The means of the different groups were compared by a one-way ANOVA Student-Newman-Keuls test. Significant differences were accepted when probability values were < 0.05.

## Results

### Ischemic postconditioning protected ischemia/reperfusion-induced brain damage, cerebral edema and neurological dysfunction

Despite the report that ischemic postconditioning protected 2 hour focal ischemia-induced brain damaged at 48 hours post reperfusion [19], it remains unclear whether ischemic postconditioning confers protection on brain damage caused by longer duration of ischemia. Thus, we compared the effect of ischemic postconditioning on brain damaged induced by focal ischemia ofvarying duration.

**Figure 1:**
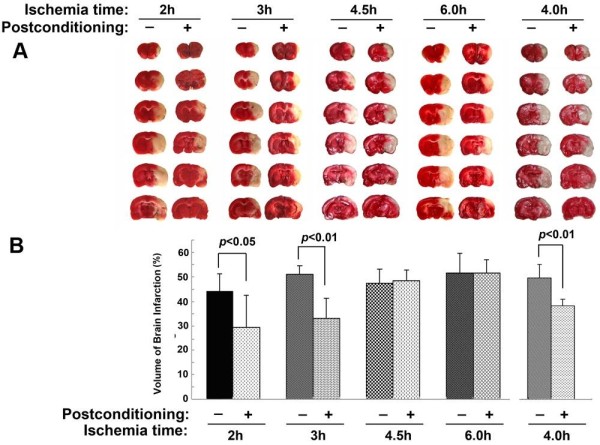
**Protection of ischemic postconditioning on cerebral infarction. (A)** Images of TTC staining. **(B)** Statistical analysis of infarction volume; (n = 6 in each group).

As shown in Figure 1, no significant difference was found between the volume of cerebral infarction produced by 23, 4.5 and 6 hours of ischemia. Ischemic postconditioning attenuated markedly the infarction size caused by 2 and 3 hours of ischemia, but not by 4.5 or 6 hours of ischemia. Further, we shortened the ischemia time to 4 hours and found ischemic postconditioning also suppressed significantly the cerebral infarction. Therefore, our results suggest that ischemic postconditioning confers protection on cerebral infarction caused by ischemia not longer than four hours.

**Figure 2:**
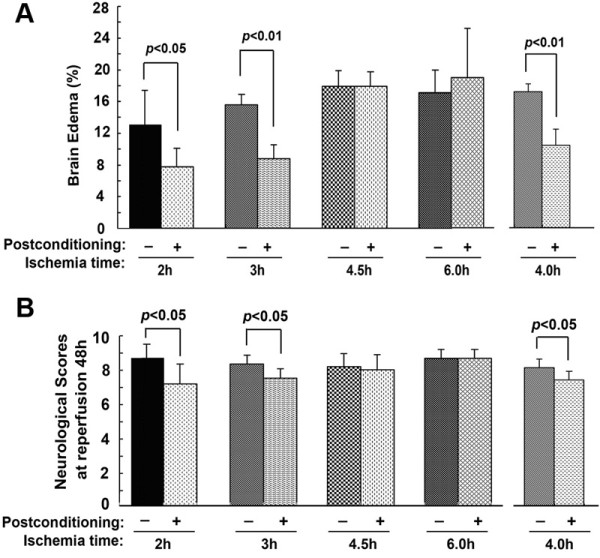
**Protective effect of ischemic postconditioning on cerebral edema and neurological function. (A)** Statistics of cerebral edema. **(B)** Statistics of neurological scores; (n = 6 in each group).

Moreover, consistent with the findings regarding cerebral infarction, neurological dysfunction and cerebraledema caused by 2, 3 and 4 hours of ischemia were all attenuated markedly by ischemic postconditioning treatment, whereas no significant changes could be found in the 4.5 and 6 hour ischemia groups (Figure 2). Thus, our results indicated that administration of ischemic postconditioning benefits brain damage caused by focal ischemia not longer than four hours.

In order to explain why ischemic postconditioning could not protect brain damage caused by 4.5 hours ischemia, we investigated the potential mechanism in the subsequent studies by comparing the differences produced by ischemia postconditioning between the rats insulted by 4.5 hours and 2 hours of ischemia.

### Ischemic postconditioning attenuated ischemia/reperfusion-induced apoptosis

**Figure 3:**
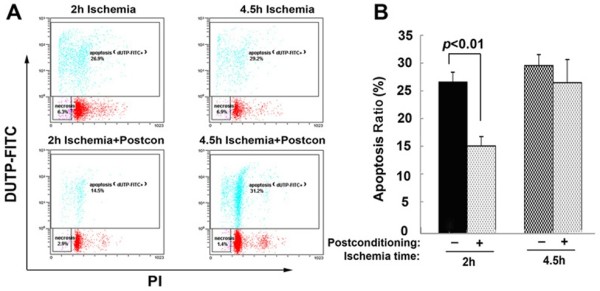
**Changes in the apoptosis between 2 hour and 4.5 hour ischemia groups treated with or without ischemic postconditioning. (A)** Representative image of flow cytometry with doubling staining with dUTP- FITC and propidium iodide (PI); **(B)** Statistical analysis of apoptotic rates; (n = 4 in each group).

Apoptosis has been found to be an important death mode of neurons during the course of ischemia and reperfusion. We thus compared the difference in the apoptotic ratio between the 2 hour ischemia group and the 4.5 hour ischemia group by using flow cytometry. Our results showed that, despite no significant difference being found in the apoptosis ratio between the 2 hour and 4.5 hour ischemia group, ischemic postconditioning markedly reduced 2 hour ischemia-induced apoptosis, while there was no difference in the apoptosis ratio between the 4.5 hour ischemia and ischemic postconditioning group (Figure 3). This indicated that the protection of ischemic postconditioning was related to inhibition of apoptosis induced by ischemia and reperfusion.

### Ischemic postconditioning suppressed the transcription and expression of toxic inflammatory cytokine IL-1β

**Figure 4:**
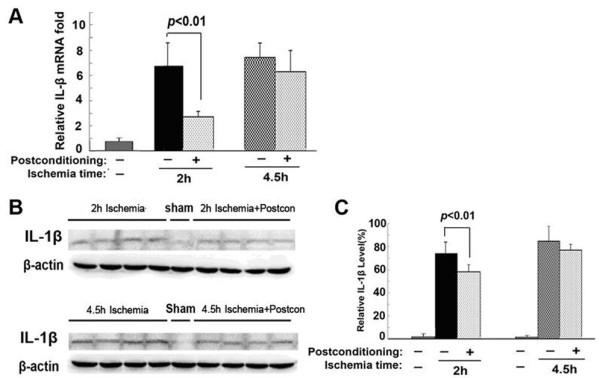
**The effect of ischemic postconditioning on the transcription and expression of IL-1β between the 2 hour and 4.5 hour ischemia groups. (A)** Statistics of the transcriptional levels of IL-1β. **(B)** Representative Western blotting images of IL-1β. **(C)** Statistics of the expressional level of IL-1β; (n = 4 in each group).

Accumulating evidence has shown that inflammation plays an important role in modulating cerebral injury and apoptosis caused by ischemia/reperfusion [20,21]; we thus examined the effects of ischemic postconditioning on the production of neurotoxic cytokine IL-1β. As Figure 4 shows, when compared with that in the sham group, either the expressional or the transcriptional level of IL-1β increased significantly in both the 2 hour ischemia group and the 4.5 hour ischemia group. However, ischemic postconditioning only suppressed markedly the elevation of IL-1β in the transcriptional and expressional levels caused by 2 hour ischemia. Thus, this indicated that the protection of ischemic postconditioning is associated with its inhibition of the production of neurotoxic cytokine IL-1β.

### Ischemic postconditioning inhibited the transcription and expression of TLR2 and TLR4

Since previous reports showed that the production of IL-1β is associated with TLR2 and TLR4 pathway [8], we thus speculated that the inhibitory effect of ischemic postconditioning on the IL-1β production might be correlated with inhibition of TLR2 or TLR4 pathway.

**Figure 5:**
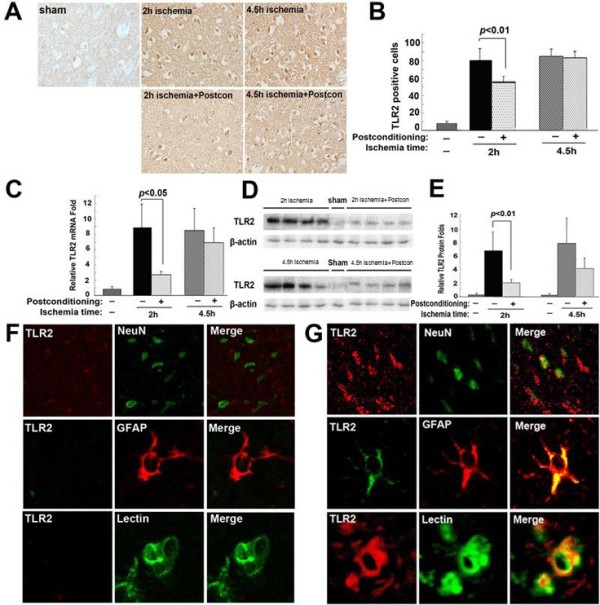
**The effect of ischemic postconditioning on TLR2 between 2 hour and 4.5 hour ischemia groups. (A)** Representative images of cells with positive immunochemical staining to TLR2, scale bar = 50 μm. **(B)** Statistics of the number of cells stained with TLR2. **(C)** Statistics of the transcriptional levels of TLR2. **(D)** Representative Western blotting images of TLR2. **(E)** Statistics of the expressional level of TLR2. **(F)** Representative images under confocal microscope in the sham group. **(G)** Representative images under confocal microscope in the ischemia group; (n = 4 in each group).

**Figure 6:**
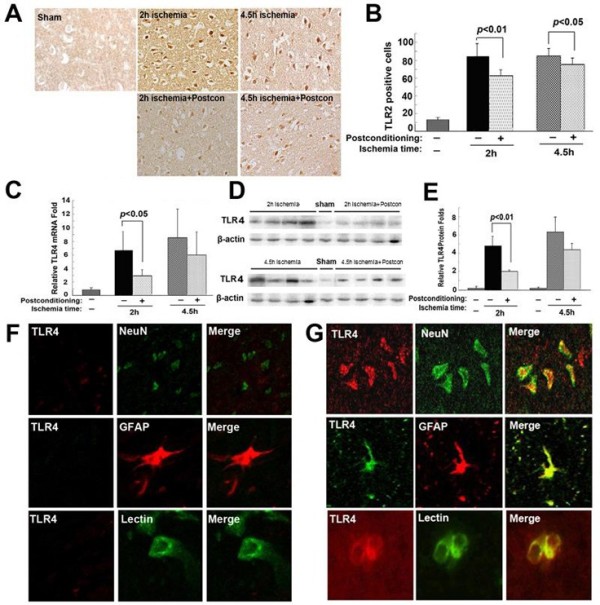
**The effect of ischemic postconditioning on TLR4 between 2 hour and 4.5 hour ischemia groups. (A)** Representative images of cells with positive immunochemical staining to TLR4, scale bar = 50 μm. **(B)** Statistics of the number of cells stained with TLR4. **(C)** Statistics of the transcriptional levels of TLR4. **(D)** Representative Western blotting images of TLR4. **(E)** Statistics of the expressional level of TLR4. **(F)** Representative images under confocal microscope in the sham group. **(G)** Representative images under confocal microscope in the ischemia group; (n = 4 in each group).

As shown in Figure 5A and B and Figure 6A and B, immunohistochemistry showed that there were significantly more TLR2 or TLR4 positive staining cells in the 2 hour or 4.5 hour ischemia group than in the sham group. When ischemic postconditioning was administered, TLR2 or TLR4 positive staining cells reduced markedly in the 2 hour ischemia group, but not in the 4.5 hour ischemia group. Since cortex contains neurons, astroglia and microglia, all of which are thought to be related to the inflammatory response [8], we used laser confocal microscopy in combination with immunohistochemical double staining to examine the location of TLR2 and TLR4 within these cells. Meanwhile, NeuN was used for detecting neurons, GFAP for astroglia and Lectin for microglia. As shown in Figures 5F and G and 6F and G, despite lower expression of TLR2 and TLR4 in the sham group, TLR2 and TLR4 were observed to be located in neurons, as well as in astroglia and microglia in the ischemia group, indicating that neurons, astroglia and microglia participate in the inflammatory response caused by ischemia and reperfusion.

Further, RT-PCR and Western blotting were used to analyze the changes in transcription and expression of TLR2 and TLR4. We found ischemic postconditioning inhibited effectively the 2 hour ischemia-induced increase of TLR2 and TLR4 in transcriptional level (Figure 5B and Figure 6B). Moreover, the higher protein levels of TLR2 and TLR4 caused by 2 hour ischemia were also inhibited. By contrast, when ischemia time was prolonged to 4.5 hours, no significant difference could be found in the transcription and expression of both TLR2 and TLR4 between the rats treated with or without ischemic postconditioning (Figures 5C and D, and 6C and D).

Therefore, our results indicated that the protection of ischemic postconditioning on brain damage caused by 2 hour ischemia/reperfusion at 48 hours is correlated with suppression of the transcription and expression of TLR2 and TLR4.

### Ischemic postconditioning mitigated transcription and expression of IRAK4

**Figure 7:**
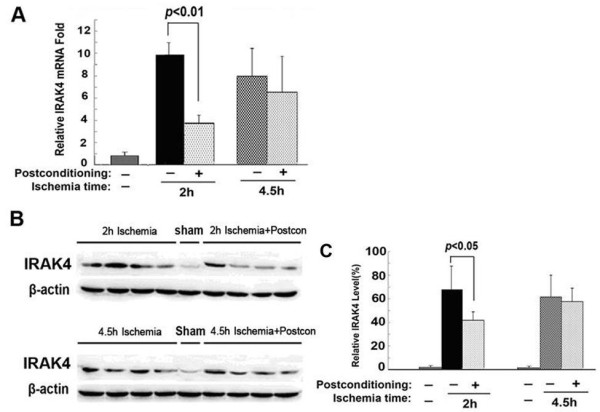
**Changes in the IRAK4 between 2 hour and 4.5 hour ischemia groups treated with or without ischemic postconditioning. (A)** Statistics of the transcriptional levels of IRAK4. **(B)** Representative Western blotting images of IRAK4. **(C)** Statistics of the expressional level of IRAK4; (n = 4 in each group).

IRAK4 (interleukin-1 receptor-associated kinase 4) is a key molecule responsible for relaying signals from TLR2 or TLR4 to NF-κB that would initiate the production of inflammatory cytokines such as IL-1β [22]; we thus compared the changes of IRAK4 transcription and expression between the rats treated with or without ischemic postconditioning. As shown in Figure 7, despite 2 hours of ischemia and 4.5 hours of ischemia leading to obvious elevation in the transcriptional and expressional level of IRAK4, only 2 hour ischemia-induced changes in IRAK4 was inhibited effectively by ischemic postconditioning. No significant influence of ischemic postconditioning on IRAK4 was found in the 4.5 hour ischemia group. Thus, this result suggested that ischemic postconditioning mitigated the transmission of inflammatory signal from TLR2 or TLR4 via inhibition of IRAK4.

## Discussion

The present study showed that five cycles of ten seconds reperfusion and ten seconds ischemia was an effective ischemic postconditioning procedure to suppress ischemia/reperfusion-induced cerebral infarction, cellular apoptosis and cerebral edema. Moreover, we found ischemic postconditioning exerted protection when ischemic period was not longer than four hours. The protective effect of ischemic postconditioning was associated with inhibition of neuroinflammation via alleviating the transcription and expression of IRAK4, TLR2 and TLR4, and attenuating the production of neurotoxic cytokine IL-1β.

Intra-arterial thrombolysis via interventional neuroradiology is an important strategy to treat the patients with cerebral artery thrombosis, but reperfusion-induced cerebral damage is the key issues influencing the therapeutic effect. For the patient without the finding of cerebral infarction on computer tomography (CT) images, six hours from the appearance of neurological symptoms is often used as a criterion to decide whether intra-arterial thrombolysis could be performed [23]. Since animal studies and clinical trials have shown ischemic postconditioning could inhibit reperfusion-induced tissue damage [19,24,25], clarifying its effects on brain damage caused by various duration of ischemia would benefit its potential administration in future clinical practice. In this study, our results showed that ischemic postconditioning conferred no protection on brain damage secondary to 6 or 4.5 hour ischemia, but protected infarct volume, cerebral edema and neurological dysfunction caused by ischemia of 4 hours or less duration. This indicated that the protection of ischemic postconditioning would be effective for 4 hours ischemic damage.

According to the time of being performed, ischemic postconditioning is classified into immediate postconditioning performed at the end of ischemia, and delayed postconditioning that is administered when blood supply has been recovered for a while. Immediate and delayed postconditioning are both proved to be protective to ischemia/reperfusion-induced brain damage, but the protective effect of immediate postconditioning is better than that of delayed postconditioning [15]. Thus, we chose to use immediate ischemic postconditioning in this study. Additionally, various ischemic postconditioning procedures consisting of different cycles of ischemia and reperfusion time were investigated in previous studies. It has been found that three cycles of 30 seconds reperfusion and 30 seconds ischemia [26], 5 minutes reperfusion and 5 minutes ischemia [27], and 30 seconds reperfusion and 10 seconds of ischemia [28] were all effective in alleviating brain damage caused by ischemia/reperfusion. Our study demonstrated that five cycles of ten seconds reperfusion and ten seconds ischemia is an effective ischemic postconditioning procedure to attenuate reperfusion-induced cerebral infarction, cerebral edema and neurological dysfunction.

To date, the protective mechanism underlying ischemic postconditioning has been investigated widely and is found to be related to multiple factors including suppression of oxidative stress, inhibition of endoplasmic stress and attenuation of apoptosis [14,19,29-31]. In this study, we found ischemic postconditioning attenuated apoptosis in the 2 hour ischemia group but not in 4.5 hour ischemia group, which could partly explain the differences in the protection generated by ischemic postconditioning between the 2 hour ischemia group and 4.5 hour ischemia group. Despite not having examined the occurrence of apoptosis in neurons or glial cells or investigating its potential mechanism, a previous study has shown that inflammatory cytokines are crucial inducers leading to apoptosis in both neurons and glial cells [32]. Moreover, ischemic postconditioning has been demonstrated to inhibit inflammation via activation of Akt-eNOS-NO-HIF pathway [14], down-regulation of the expression of Egr-1 (early growth response-1) and RGMa (repulsive guidance molecule A) [13,15], suppression of TGF-β1/phospho-Smad2 pathway [33], and blocking the increases of microglia, macrophages, CD4 T cells and CD8 T cells as well as B lymphocytes in damaged tissue [11]. However, it is still unclear whether the inhibitory effect of ischemic postconditioning on inflammation is via modulating TLRs.

IL-1β is an interesting neurotoxic cytokine possessing autocrine-like function and promotes its own secretion under ischemic stimuli, which directly induces neuronal apoptosis and enhances the expression of chemokines within microglia and astrocyte [8]. It was reported previously that ischemic postconditioning could inhibit the production of neurotoxic cytokines IL-1β, as well as IL-6, IL-8 and TNF-α [13,15]. Our study showed that ischemic postconditioning inhibited the production IL-1β in the 2 hour ischemia group, not in 4.5 hour ischemia group, which might be the reason underlying the differential protection of ischemic postconditioning between the 2 hour ischemia group and 4.5 hour ischemia group.

Given that TLR2 and TLR4 play an active role in modulating the production of neurotoxic cytokines during the course of brain damage caused by ischemia and reperfusion [34], we also comparedthe changes in TLR2 and TLR4 between the 2 and 4.5 hour ischemia group when ischemic postconditioning was administered. Our results showed that ischemic postconditioning not only inhibited 2 hour ischemia-induced abnormal transcription and expression of TLR2 and TLR4, but also suppressed the transcription and expression of IRAK4, a protein mediating signals from TLR2 or TLR4 to NF-κB to initiate the transcription of IL-1β [22,35]. Similarly, attenuation of inflammation by inhibition of TLR2, TLR4 and IRAK4 has also been reportedby other researchers. Ziegler *et al*. reported that blocking TLR2 protected against accumulation of inflammatory cells and neuronal injury in experimental stroke [36]. Yao *et al*. found that neutralization of TLR4 in primary cultured microglia attenuated hypoxia-induced expression of TNF-α and IL-1β in BV-2 cells [37]. Wang *et al*. revealed that IRAK4 knockout mice were severely impaired in signaling and cellular responses to IL-1, IL-18, and most TLR ligands [22]. Therefore, we think that the protection of ischemic postconditioning on ischemia/reperfusion-induced brain damage is closely associated with inhibition of neuroinflammation via down-regulation of the transcription and expression of IRAK4, TLR2 and TLR4.

In conclusion, we demonstrate in this study that ischemic postconditioning is effective up to 4 hours after ischemia. The protection of ischemic postconditioning on ischemia/reperfusion-induced cerebral infarction, cerebral edema and neurological dysfunction is associated with its attenuation of apoptosis, and inhibition of neuroinflammation via modulation of the TLR2 and TLR4 pathways.

## Abbreviations

TLR: Toll-lile receptors; TLR2: Toll-like receptor 2; TLR2: Toll-like receptor 2; IRAK4: Interleukin-1 receptor-associated kinase 4; MCAO: Middle cerebral artery occlusion; CCA: Common carotid artery; HSPs: Heat-shock proteins; HMGB1: High mobility group box 1; RGMa: Repulsive guidance molecule A.

## Competing interests

The authors declare that they have no competing interests.

## Authors’ contributions

YW, PG and YZ designed the study. YW, LY, HZ and TL performed the experiments. YW and PG analyzed data and wrote the manuscript. YW, PG and CW revised the manuscript. All authors have read and approved the final version of the manuscript.
